# Development of Bioinspired Gelatin and Gelatin/Chitosan Bilayer Hydrofilms for Wound Healing

**DOI:** 10.3390/pharmaceutics11070314

**Published:** 2019-07-04

**Authors:** Itxaso Garcia-Orue, Edorta Santos-Vizcaino, Alaitz Etxabide, Jone Uranga, Ardeshir Bayat, Pedro Guerrero, Manoli Igartua, Koro de la Caba, Rosa Maria Hernandez

**Affiliations:** 1 NanoBioCel Group, Laboratory of Pharmaceutics, School of Pharmacy, University of the Basque Country (UPV/EHU), Paseo de la Universidad 7, 01006 Vitoria-Gasteiz, Spain; 2 Biomedical Research Networking Centre in Bioengineering, Biomaterials and Nanomedicine (CIBER-BBN), 01006 Vitoria-Gasteiz, Spain; 3 BIOMAT Research Group, Chemical and Environmental Engineering Department, Engineering College of Gipuzkoa, University of the Basque Country (UPV/EHU), Plaza de Europa 1, 20018 Donostia-San Sebastián, Spain; 4 Plastic & Reconstructive Surgery Research, Division of Musculoskeletal & Dermatological Sciences, School of Biological Sciences, University of Manchester, M13 9PL Manchester, UK

**Keywords:** wound healing, gelatin, chitosan, bilayer dressing, ex vivo model, human skin

## Abstract

In the current study, we developed a novel gelatin-based bilayer wound dressing. We used different crosslinking agents to confer unique properties to each layer, obtaining a bioinspired multifunctional hydrofilm suitable for wound healing. First, we produced a resistant and non-degradable upper layer by lactose-mediated crosslinking of gelatin, which provided mechanical support and protection to overall design. For the lower layer, we crosslinked gelatin with citric acid, resulting in a porous matrix with a great swelling ability. In addition, we incorporated chitosan into the lower layer to harness its wound healing ability. FTIR and SEM analyses showed that lactose addition changed the secondary structure of gelatin, leading to a more compact and smoother structure than that obtained with citric acid. The hydrofilm was able to swell 384.2 ± 57.2% of its dry weight while maintaining mechanical integrity. Besides, its water vapour transmission rate was in the range of commercial dressings (1381.5 ± 108.6 g/m^2^·day). In vitro, cytotoxicity assays revealed excellent biocompatibility. Finally, the hydrofilm was analysed through an ex vivo wound healing assay in human skin. It achieved similar results to the control in terms of biocompatibility and wound healing, showing suitable characteristics to be used as a wound dressing.

## 1. Introduction

Bioinspired bilayer dressings are a promising approach for wound healing, since they are able to provide a structure that mimics the bilayer structure of skin, with an upper protective layer resembling the epidermis, and a thicker flexible lower layer like the dermis. Taking that into account, the dense upper layer is designed to cover the wound, and to give mechanical strength to the dressing. In addition, it needs to control moisture transmission to prevent fluid loss and dehydration, while allowing exudate removal. Furthermore, the upper layer prevents bacterial penetration and thus wound infection. On the other hand, the lower layer needs to be a porous or sponge like structure able to absorb wound exudate and smoothly adhere to the wet wound bed to accommodate newly formed tissue [1,2,3].

Usually, each layer consists of a different polymer. However, depending on its crosslinking degree and nature, gelatin is able to exhibit the characteristics needed to constitute both layers. Gelatin is a natural polymer derived from collagen. Compared to the latter, it is more inexpensive and less antigenic, since it is partially denatured [4,5]. Moreover, it has been extensively used in medical and pharmaceutical applications due to its biocompatibility and biodegradability, and it has been recognized as GRAS (Generally Recognized As Safe) by the Food and Drug Administration (FDA) [6]. In addition, it has multiple characteristics that make it a suitable option to develop wound dressings, such as an excellent ability to form films and hydrogels. Secondly, gelatin chains contain arginine-glycine-aspartic (RGD) motifs, an important sequence in the promotion of cell adhesion, which gives gelatin an improved biological behaviour in comparison to other polymers [7]. Lastly, the flexible amino acidic structure of gelatin presents diverse free functional groups that allow chemical conjugation and thus the modification of the gelatin structure.

Gelatin presents an excellent ability to absorb large volumes of water and thereby create hydrogels. Hydrogels have been widely used for wound healing since they present a variety of advantages for their use as dressings. First, they provide fluid absorption or wound hydration, since they can absorb or donate water depending on their environment. In addition, hydrogels are also able to cool wound surface and relieve pain. This is reinforced by the fact that they have permeability to water vapour and oxygen without water leaks. Finally, they are suitable for unusual wound shapes due to their jelly-like nature [8].

When it comes to developing a gelatin wound dressing, crosslinking is essential to increase gelatin’s mechanical properties and reduce its solubility or degradation rate in an aqueous environment. Moreover, depending on the crosslinking degree and nature (e.g., sensitivity of the bound to hydrolytic degradation), gelatin can adopt physical forms ranging from amorphous gels to semi-stiff sheets. Therefore, a correct choice of crosslinking agents and protocols may enable gelatin to constitute both layers of a bilayer dressing [8]. Crosslinking methods include physical, biological and chemical methods [9]. Among chemical methods, aldehydes such as glutaraldehyde are the most commonly used reagents, although unreacted toxic products can be trapped into the hydrogel [10]. In this sense, other crosslinkers have gained importance due to their biocompatibility including genipin, lactose and citric acid. The crosslinking reaction differs with each of them; genipin reacts with the amine groups of the gelatin to form a heterocyclic compound [11]; secondly, lactose leads a non-enzymatic glycation of the protein chains known as Maillard reaction [12]; and finally, citric acid reacts with the amine group of the gelatin [13]. Thereby, with each crosslinker the stability and strength of the bound differs, leading to gelatin with unique properties.

On the other hand, in order to accelerate wound healing, natural polymers such as chitosan can be added to the dressings. Chitosan is a biocompatible and biodegradable polymer derived from chitin, a compound of the exoskeleton of insects and crustaceans [14]. It has shown to accelerate wound healing through diverse mechanisms: it presents antibacterial and haemostatic activity, acting in the early phases of healing [15]. In addition, it promotes the migration of polymorphonuclear neutrophils and the activity of macrophages. Lastly, it enhances the formation of the granulation tissue by inducing the proliferation of dermal fibroblasts [16].

The efficacy of wound dressings is usually tested in wound healing assays conducted in animals. However, animal models do not accurately mimic the structure of human skin or the human process of wound healing [17]. Moreover, ethical concerns discourage their use [18]. An alternative to reduce the use of animal experimentation are in vitro and ex vivo models. Among in vitro models single cell culture, co-culture and organotypic culture can be distinguished. Single monolayer cell cultures of keratinocytes, fibroblasts or endothelial cells are used to analyse basic physiological processes through scratch, chemotaxis or tube formation assays [19,20]. Co-cultures of keratinocytes and fibroblast using transwell systems are used to analyse the interaction between those cell types [21]. Finally, organotypic culture consists in seeding keratinocytes on top of a collagen gel containing fibroblasts. Those systems have been used to investigate scar pathology [22]. Nevertheless, the use of those models is limited due to the lack of extracellular matrix (ECM) components and the native skin structure. To overcome those limitations, whole skin biopsies in culture can be used. Those ex vivo models mimic normal skin more closely, and they enable an evaluation of wound healing process both in implicated cells and in the surrounding ECM components. These models have already been established as a useful tool to evaluate wound healing. For that purpose, partial or full-thickness wounds are made in the centre of the biopsies, and wound healing assessed after an incubation period [23,24,25].

Accordingly, the aim of this study was to develop a bioinspired gelatin and gelatin/chitosan bilayer wound dressing. In order to regulate the crosslinking degree and nature of each layer, and thereby their properties, different crosslinking agents were used. The upper layer was crosslinked with lactose, obtaining a rigid layer able to provide mechanical strength and protection to the dressing. The lower layer was crosslinked with citric acid, obtaining a hydrogel able to absorb a larger volume of water and degrade. In addition, chitosan was added to the lower layer to enhance its wound healing properties. Firstly, both monolayer hydrofilms and the resulting bilayer hydrofilms were characterised in terms of protein structure, morphology, structure, swelling ability and oclussivity. Additionally, cytotoxicity studies were performed following the ISO 10993-5:2009 guideline to evaluate the safety of the developed formulation. Finally, the biocompatibility and efficacy of the dressing were evaluated using an ex vivo model of a partial thickness wound.

## 2. Materials and Methods

### 2.1. Hydrofilms Production

Type A fish gelatin, with a bloom value of 240 and average molar mass of 125–250 kDa (Healan Ingredients, North Newbald, UK), was used as the main component of film forming formulations. Lactose and anhydrous citric acid (Panreac, Barcelona, Spain) were used as crosslinkers. Chitosan, with a deacetylation degree higher than 75% and molecular weight of 375 kDa (Sigma Aldrich, Madrid, Spain) was used as a bioactive compound.

On the one hand, gelatin films crosslinked with lactose (Lac) were prepared. Hence, 5-g gelatin and 20 wt % lactose (on gelatin dry basis) were dissolved in 100 mL distilled water for 30 min at 80 °C under continuous stirring to obtain a good blend. After that, pH was adjusted to 10 with NaOH (0.1 M), and the solution was maintained at 80 °C for other 30 min under stirring. Finally, 17 mL of the film forming solution were poured into each Petri dish and left drying 48 h at room temperature to obtain films. The films peeled from the Petri dishes were heated at 105 °C for 24 h to obtain monolayer hydrofilms crosslinked with lactose (mHF-Lac).

On the other hand, gelatin films crosslinked with citric acid (CA) and containing chitosan (Chit) were prepared. Firstly, 10 wt % (on gelatin basis) citric acid solutions were prepared. Then, 9 wt % chitosan (on gelatin basis) was dissolved in 100 mL of citric acid solution and it was maintained under continuous stirring for 30 min. After that, 5-g gelatin were added and the resultant solution was heated at 80 °C for 30 min and stirred at 200 rpm. Then, pH was adjusted to 4.5 using NaOH (0.1 M), and the solution was stirred for other 30 min at 80 °C and 200 rpm. Finally, 17 mL of film forming solution were poured into each Petri dish and left drying 48 h at room temperature to obtain monolayer hydrofilms crosslinked with citric acid and containing chitosan (mHF-CA+Chit). Hydrofilms without chitosan were also prepared (mHF-CA).

**Figure 1:**
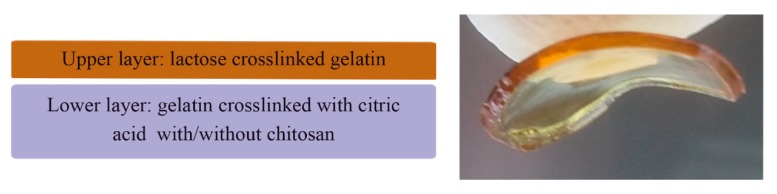
Scheme and photograph of gelatin bilayer films.

Furthermore, bilayer hydrofilms were prepared using mHF-Lac as the upper layer and mHF-CA or mHF-CA+Chit as the lower layer, as shown in Figure 1. Both layers were glued together spraying ethanol on them and letting them air-dry.

All films were conditioned in an ACS Sunrise 700 V bio-chamber (Alava Ingenieros, Madrid, Spain) at 25 °C and 50% relative humidity before testing. The developed formulations are summarised in Table 1.

### 2.2. Fourier Transform Infrared (FTIR) Spectroscopy

FTIR spectra were recorded on a Nicolet 380 FTIR spectrometer equipped with horizontal attenuated total reflectance (ATR) crystal (ZnSe). The spectra were collected in absorbance mode on sample films. The measurements were recorded between 4000 and 800 cm^−1^. A total of 32 scans were made for each sample, at 4 cm^−1^ resolution. All spectra were smoothed using the Savitzky–Golay function. Second-derivative spectra of the amide region were used at peak position guides for the curve fitting procedure, using OriginPro 9.1 software (OriginLab, Northampton, MA, USA).

### 2.3. Scanning Electron Microscopy (SEM)

The morphology of the cross-section of the films was visualized using a Hitachi S-4800 scanning electron microscopy (Hitachi, Ibaraki, Japan). The cross-section was prepared using mechanical means like conventional cutter. Then, samples were mounted on a metal stub with double-side adhesive tape and coated under vacuum with gold, using a JEOL fine-coat ion sputter JFC-1100 (Izasa, Madrid, Spain) in an argon atmosphere prior to observation. All samples were examined using an accelerating voltage of 15 kV.

### 2.4. Water Uptake

A water uptake curve was determined weighing the water uptake of the hydrofilms at different time points. Firstly, dry samples of each formulation were cut in discs of 12 mm in diameter. Then, discs were weighed and soaked in 1 mL of Phosphate Buffered Saline (PBS) at 4 °C (pH 7.4, Gibco^®^ Life technologies, Madrid, Spain). At various time points (30 min, 2 h, 24 h, 48 h and 72 h) hydrofilms were removed from the PBS, the excess of liquid wiped out with a filter paper, and the wet weight determined. The percentage of water uptake (WU) was calculated through the following equation (Equation (1)):

(1) $$WU\left(\%\right)=\frac{W\;-\;W_{0}}{W_{0}}\times 100$$

Where, W is the weight of the wet sample at each time point and W_0_ is the weight of the dry samples.

### 2.5. Hydrolytic Degradation

First, hydrofilms were cut in discs of 12 mm in diameter, weighed and immersed in PBS at 4 °C for 72 h (n = 3). Then, they were washed with MilliQ water and left to dry for 7 days. Once discs were completely dried, they were weighed again and the percentage of the remaining weight was calculated using the following equation (Equation (2)). Three independent experiments were performed.

(2) $$\mathrm{Remaining}\;\mathrm{weight}\;\left(\%\right)=\frac{\mathrm{Dry}\;\mathrm{weight}\;}{\mathrm{Initial}\;\mathrm{weight}}\times 100$$

### 2.6. Water Vapour Transmission Rate (WVTR)

In order to analyse the ability of hydrofilms to regulate moisture, WVTR was calculated. The WVTR of the mHF-CA and mHF-CA+Chit were not measured since once wet they lost the structural stability needed to seal the Franz diffusion cell due to their great ability to swell water. The WVTR was quantified following the method described by Etxabide et al. [26]. Briefly, the receptor compartment of a Franz diffusion cell was completely filled with Milli-Q water and its receptor arm was sealed with parafilm. Then, hydrofilm discs slightly bigger than the aperture between compartments (10 mm) were placed between them, to make the hydrofilm the only way for water vapour to leave the system. The complete assembly was weighed at the beginning of the study and after a 48 h incubation at room temperature. The WVTR was calculated using the following equation (Equation (3)):

(3) $$\mathrm{WVTR}=\frac{M_{1}-M_{0}}{A\times T}$$

where M_0_ is the weight of the assembly at the beginning of the assay, M_1_ is its weight after the incubation time, A is the exposure area (0.79 cm^2^) and T is the exposure time (2 days).

### 2.7. Cytotoxicity Study

Cytotoxicity studies were performed using the L-929 fibroblasts (ATCC, Manassas, VA, USA), since it is the cell line recommended by the ISO 10993-5:2009 guideline for biological evaluation of medical devices. Cells were cultured on Eagle’s Minimum Essential Medium (EMEM; ATCC, Manassas, VA, USA) supplemented with 10% (*v*/*v*) inactivated Horse Serum and 1% (*v*/*v*) penicillin-streptomycin, and incubated at 37 °C in a humidified incubator with a 5% CO_2_ atmosphere. Cell passages were performed every 2–3 days depending on cell confluence.

Indirect cytotoxicity was assessed incubating cells with the extracted medium of the hydrofilms. Firstly, hydrofilms were sterilized by impregnating both sides with 70% ethanol and exposing them to UV light for 20 min. Subsequently, the hydrofilms were divided into two groups that received a different processing method. Hydrofilms in the first group were dialyzed in 1 L of Milli-Q water for 72 h and then they were maintained in culture medium for another 24 h to achieve an osmotic equilibrium. The second group was simply hydrated for 15 min in culture medium. Afterwards, 16-mm discs of each hydrofilm were incubated with 0.5 mL of culture medium for 24 h at 37 °C to obtain the released medium.

Meanwhile, cells were seeded on 96-well plates at a density of 5000 cell/well and incubated overnight to allow cell attachment. Then, the medium was replaced by the released medium of the hydrofilms, although some wells were replaced by fresh culture medium to be used as controls (n = 3). After 24 h of incubation, cell viability was assessed using the CCK-8 colorimetric assay (Cell Counting Kit-8, Sigma-Aldrich, Saint Louise, MO, USA). Briefly, 10 µL of the CCK-8 reagent were added to the cells and incubated for 4 h. Then, the absorbance of the wells was read at 450 nm, using 650 nm as reference wavelength (Plate Reader Infinite M200, Tecan, Männedorf, Switzerland). The absorbance value was directly proportional to the number of living cells in each well. Results were given as the percentage of living cells regarding to control. Three independent experiment were performed.

### 2.8. Ex Vivo Assay

#### 2.8.1. Ex Vivo Assay Procedure

Explants were obtained from three healthy patients undergoing routine elective surgery, demographic data are summarised in Table 2. Ethical approval for this study was provided by the North-West of England research ethics committee (11/NW/0683) 2017.

Explants were washed several times in PBS (Sigma-Aldrich, Gillingham, UK) and soaked in Dulbecco’s modified Eagle’s medium (DMEM, D6429-500 mL, Sigma-Aldrich, UK) supplemented with 100 UI/mL of penicillin-streptomycin (Sigma-Aldrich, Gillingham, UK), 0.1% (*v*/*v*) insulin (Sigma-Aldrich, Gillingham UK) and 0.001% (*w*/*v*) hydrocortisone (Sigma-Aldrich, Gillingham, UK).

**Figure 2:**
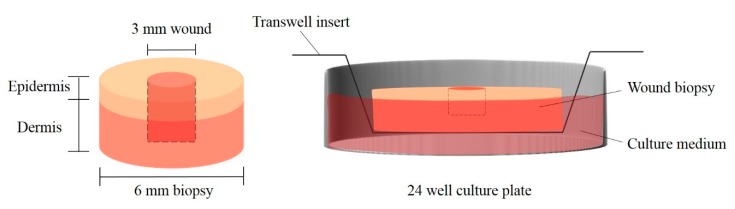
Ex vivo assay scheme.

The ex vivo assay was conducted modifying the method described by Hodgkinson et al. and Mendoza-Garcia et al., a scheme of the method is showed in Figure 2 [27,28]. Explants were cut in 6 mm in diameter biopsies using a punch biopsy (Kai Europe, GmbH, Solingen, Germany) and allowed to equilibrate overnight in culture medium at 37 °C in a humidified incubator with a 5% CO_2_ atmosphere. Afterwards, full thickness excisional wounds (donut-shaped model) of 3 mm in diameter were made in the centre of the samples. Biopsies were then transferred to transwell inserts (Corning, New York, NY, USA) and cultured in 24 well plates. The dermis was immersed into supplemented DMEM medium and the epidermis was exposed to liquid-air interface. However, upon being transferred to transwells, the dermis surrounding the wounds expanded into them, filling the void partially, which made the model behave as a partial thickness wound model thenceforth.

Then, biopsies were divided into three groups (n = 9, three from each patient): (i) the control group did not receive any treatment; (ii) a disc of 6 mm diameter of bHF+Chit was applied to the biopsies; and (iii) a disc of 6 mm diameter of mHF-Lac was applied to the biopsies. Before their application, the hydrofilms were sprayed with ethanol in both sides, kept under UV light for 30 min and hydrated for 15 min. Hydrofilms were applied on top of the wounds, letting the lower layer be in close contact with the wound bed, which was composed of the dermis inside the wounds. Treatments were changed on day 4. Biopsies were incubated for 8 days at 37 °C in a humidified incubator with a 5% CO_2_ atmosphere and culture medium was changed every day.

#### 2.8.2. Lactate Deshydrogenase (LDH) Assay

On days 4 and 8, 50 µL of the medium were collected from the wells to perform a LDH (lactate dehydrogenase) assay (Pierce™ LDH Cytotoxicity Assay Kit, ThermoFisher Scientific Inc., Waltham, MA, USA), in order to assess the viability of the biopsies. The LDH assay was conducted according to the manufacturer instructions. Briefly, 50 µL of lysis buffer was added to an extra biopsy included to be used as the control of this assay and the mixture was incubated for 45 min. Then, 50 µL of each biopsy (including the one treated with the lysis buffer) were transferred into a 96 well plate and 50 µL of the LDH reagent were added to the wells. The mixture was incubated for less than 30 min at room temperature and protected from light. The reaction was stopped with 50 µL of the stop reagent and the absorbance was read at 492 nm, using 680 nm as reference wavelength. Cell viability was expressed using Equation (4).

(4) $$\mathrm{Cell}\;\mathrm{viability}\;\left(\%\right)=\frac{A_{L}-A_{S}}{A_{L}}\times 100$$

where, A_L_ is the absorbance of the samples incubated with the lysis buffer and A_S_ is the absorbance of the tested samples.

#### 2.8.3. Tissue Processing

On days 1 and 8, the treatments were removed from the wounds, and wounds were gently washed by immersing them in PBS samples were processed to assess wound healing. Then, tissues biopsied were fixed in 3.7% paraformaldehyde for 24 h, then they were embedded in paraffin and sectioned in layers of 5 µm in thickness. Three tissue sections of each biopsy were used for histological analyses.

Stained images were acquired in a 3D-Histech Pannoramic-250 microscope slide-scanner using a 20x objective (Zeiss, Oberkochen, Germany). Snapshots of the slide-scans were taken using the Case Viewer software (3D-Histech, Budapest, Hungary).

Regarding histological analyses, tissue sections were stained with Hematoxylin-Eosin (H&E) to evaluate wound closure. Briefly, wound closure was determined measuring the epithelial gap between epidermal tongues using 4X magnification snapshots taken with the CaseViewer software. Then, the values were normalized to the initial wound measurement on day 0.

In addition, immunohistochemical analyses were conducted using antibodies against α-SMA (1:250 dilution, ab5696, Abcam, Cambridge, UK), proliferating cell nuclear antigen (PCNA) (1:10000, ab181797, Abcam, Cambridge, UK), cytokeratin 10 (1:5000, ab 76318, Abcam, UK) and cytokeratin 14 (1:250, Abcam, Cambridge, UK). Briefly, sections were deparaffinised, and after the blocking step, they were incubated with the primary antibodies overnight at 4 °C. Thereafter, sections were revealed using the ImmPRESS™ Peroxidase Detection Kit (Vector Laboratories LTD., Peterborough, UK) and ImmPACT DAB substrate (Vector Laboratories LTD., Peterborough, UK) following the manufacturer’s instructions. Finally, sections were counterstained with hematoxylin (Vector Laboratories LTD., Peterborough, UK), dehydrated and mounted. The stained area in each tissue section or the positive cell numbers were measured using the Tissue Studio analysis software (Definiens AG, München, Germany).

The results obtained in the sections stained with antibodies against α-SMA, cytokeratin 10 and cytokeratin 14 were expressed as the percentage of stained area in the tissue section, while the results of the sections stained with antibodies anti-PCNA were expressed as the number of positive cells. Thereafter, in order to reduce interindividual variability, results were normalized to the percentage of the values obtained on day 0 for each patient.

### 2.9. Statistical Analysis

Results were expressed as the mean ± standard deviation (SD), except the results of the histological and immunohistochemical analysis that were expressed as the mean ± standard error of the mean. Results were analysed through one-way ANOVA test for multiple comparisons. Based on the Levene test for the homogeneity of variances, Bonferroni or Tamhane post-hoc were applied. All the statistical tests were performed using SPSS 22.0.01 (SPSS^®^, INC., Chicago, IL, USA).

## 3. Results

### 3.1. Film Structure

**Figure 3:**
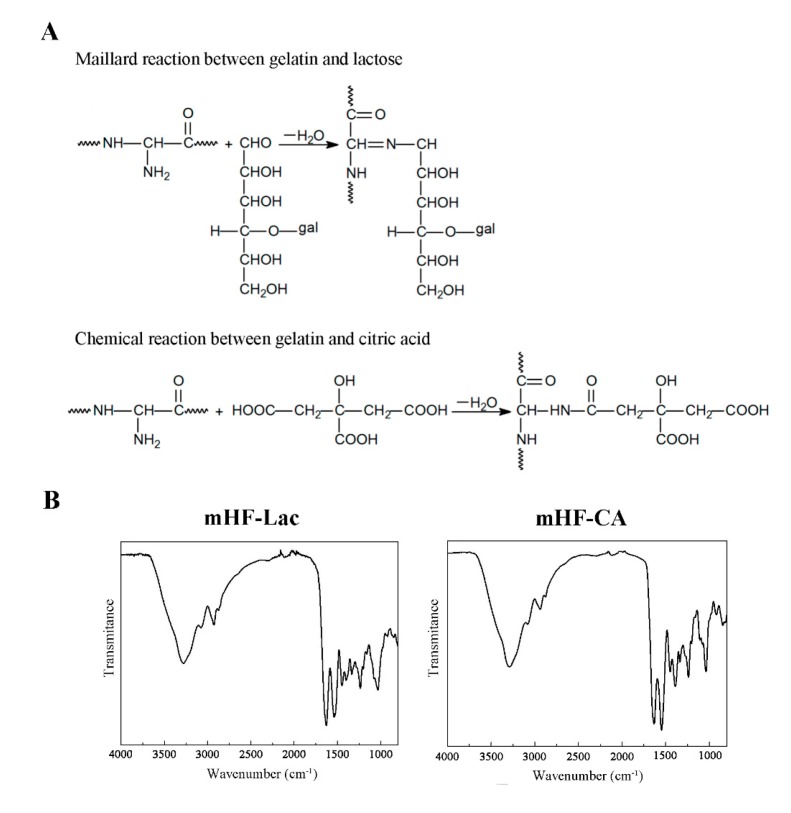

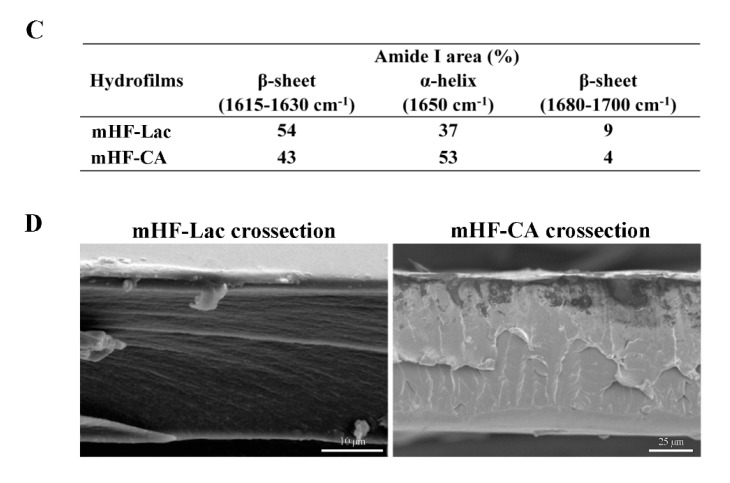
Gelatin crosslinking. (**A**) Representation of the early stage of Maillard reaction between gelatin and lactose (gal=galactose) and the chemical reaction between gelatin and citric acid. (**B**) Fourier-transform infrared (FTIR) spectra of mHF-Lac and mHF-CA. (**C**) Protein conformation in gelatin films crosslinked with lactose or citric acid. (**D**) Scanning electron microscopy (SEM) analysis of mHF-Lac and mHF-CA cross-section.

In this work, the structure and thus, the physical performance of gelatin films was modified by crosslinking. One of the hydrofilms was crosslinked by means of a non-enzymatic glycation (Maillard reaction) between gelatin and lactose (mHF-Lac); and the other hydrofilm was crosslinked by the amide linkage formed between the amino groups of gelatin and the carboxylic groups of citric acid (mHF-CA), as shown in Figure 3A.

These facts were confirmed analysing the effect of lactose and citric acid in protein structure by FTIR. The most relevant peaks in FTIR spectra were related to the peptide bonds of the protein: C=O stretching at 1630 cm^−1^ (amide I), N-H bending at 1530 cm^−1^ (amide II), and C–N stretching at 1230 cm^−1^ (amide III). The broad band observed in the 3500–3000 cm^−1^ range is attributable to free and bound –OH and –NH- groups, which are able to form hydrogen bonding with the carbonyl group of the peptide linkage in the protein. As shown in Figure 3B, the most notable difference found among mHF-CA and mHF-Lac can be observed in the relative intensity between amide I and amide II, indicating the change of gelatin structure as a consequence of the crosslinking.

In addition to the qualitative analysis carried out above, the band corresponding to amide I was used for the quantitative analysis of the changes in the secondary structures of protein backbone due to crosslinking. In particular, the changes in the intensity of the bands assigned to α-helix/unordered structures (1650 cm^−1^) and to β-sheets (1615–1630 cm^−1^ and 1680–1700 cm^−1^). This quantitative analysis is shown in Figure 3C. The area of the two bands related to β-sheets (1625 and 1689 cm^−1^) was higher for mHF-Lac than for mHF-CA, while the area related to α-helix/unordered structures was lower, revealing that gelatin crosslinked with lactose presented a more ordered structure than gelatin crosslinked with citric acid.

This change of structure was also observed by SEM analysis of the film cross-section. As can be observed in Figure 3D, a more homogeneous, compact and smoother stratified structure was found for the mHF-Lac.

### 3.2. Water Uptake

**Figure 4:**
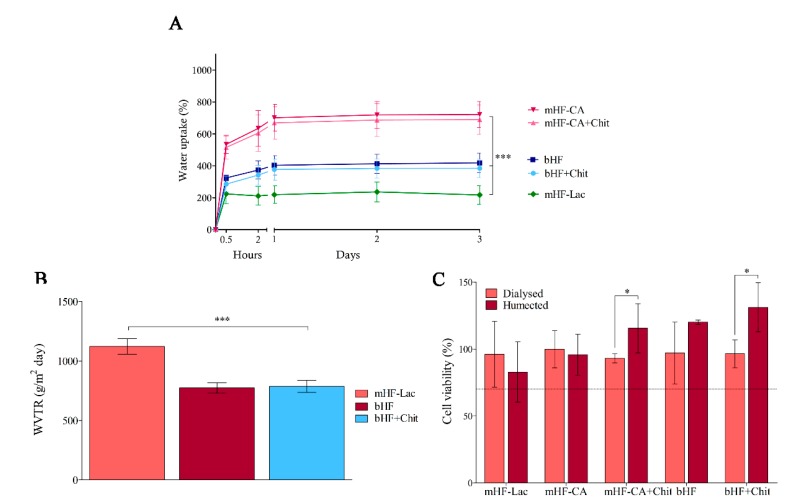
Hydrofilms characterisation. (**A**) Swelling curve. The percentage of water uptake regarding dry weight of the hydrofilms at different time points. ***p < 0.001 among mHF-Lac, bilayer hydrofilms (bHF and bHF+Chit) and monolayer hydrofilms crosslinked with citric acid (mHF-CA and mHF-CA+Chit). (**B**) Water vapour transmission rate (WVTR). Graphical representation of the WVTR of the hydrofilms ***p < 0.001 comparing mHF-Lac with bHF and bHF+Chit. Results are given as mean ± SD. (**C**) Cytotoxicity assay. Cell viability after incubating fibroblasts with the release medium of hydrofilms. *p < 0.05 comparing dialysed and humected formulations. Results are shown as mean ± SD.

As depicted in Figure 4A, the swelling curve demonstrated the same tendency in almost every time point tested. The hydrofilms with the greatest swelling ability were mHF-CA and mHF-CA+Chit, showing a swelling ability of about 700% in equilibrium. On the contrary, mHF-Lac absorbed 4–5-fold less water than the previous ones, with a swelling value about 200 % (***p < 0.001). Finally, the bilayer hydrofilms presented intermediate values. bHF+Chit showed a swelling of 384.2 ± 57.2% and bHF a value of 418.8 ± 60.8% at 72 h (***p < 0.001 against mHF-Lac, mHF-CA and mHF-CA+Chit). The addition of chitosan did not produce any effect in the ability to absorb water, since no differences were observed among similar hydrofilms with and without chitosan, i.e., bHF and bHF+Chit or mHF-CA and mFH-CA+Chit.

### 3.3. Hydrolytic Degradation

In order to assess the degradation of the hydrofilms in an aqueous environment, they were immersed in PBS for 72 h. Dry hydrofilms were weighed at the beginning and at the end of the study, and all the formulations had a remaining weight of about 96% of their initial weight.

### 3.4. WVTR

The occlusivity of the hydrofilms was assessed trough WVTR. As observed in Figure 4B, there was no differences between bilayer hydrofilms (bHF and bHF+Chit) as they showed similar values, 773.7 ± 43.4 g/m^2^·day and 787.0 ± 50.9 g/m^2^·day respectively. On the contrary, mHF-Lac showed a less occlusive character, as it achieved a significantly higher WVTR value, 1122.5 ± 64.8 g/m^2^·day.

### 3.5. Cytotoxicity Assay

The cytotoxicity assay was performed to evaluate the effect of the hydrofilms on cells. Two processing methods were used before the assay. The first method was a 72-h dialysis to remove the unreacted crosslinker and the rest of compounds released from the hydrofilms. The second method consisted in hydrating the hydrofilms for 15 min.

Results of the CCK-8 are shown in Figure 4C, proving that none of the hydrofilms was cytotoxic, since fibroblasts incubated with the released medium of the hydrofilms maintained their viability above 70%. The only difference observed between the processing methods was found in the formulations containing chitosan, where a statistically significantly higher viability was observed with humected hydrofilms than with dialysed hydrofilms.

### 3.6. Ex Vivo Assay

In order to assess the efficacy of the hydrofilms on wound healing, an ex vivo assay in human skin was performed. Skin explants with partial thickness wounds of 3 mm were cultured for eight days in transwell culture inserts.

Taking the results obtained in the previous assays into account, bHF+Chit and mHF-Lac were the only hydrofilms analysed ex vivo. Among bilayer hydrofilms, only bHF+Chit was evaluated, due to the wound healing activity of chitosan. In addition, among the monolayer hydrofilms, only mHF-Lac was tested, since wet mHF-CA and mHF-CA+Chit did not have enough structural stability to be used as dressings on their own. Non treated skin explants were used as a control.

**Figure 5:**
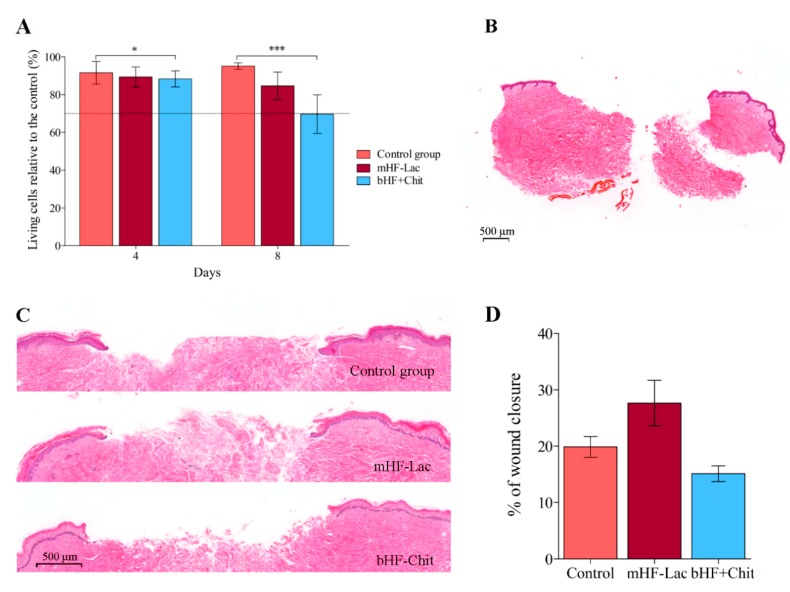
Ex vivo assay wound healing results. (**A**) LDH assay of the ex vivo assay. The results were represented as the viability of the three groups on different time points. *p < 0.05 comparing the control group and the group treated with bHF+Chit, on day 4; ***p > 0.001 comparing each group with the other two, on day 8. (**B**) Representative image of a tissue section stained with H&E on day 0. The scale bar indicates 500 µm. (**C**) Histological images of tissue sections of each group on day 8, processed with H&E. The scale bar indicates 500 µm. (**D**) Percentage of wound closure on day 8. Results are shown as the mean ± standard error of the mean. Results are shown as the mean ± standard error of the mean.

On days 4 and 8 of the study, a LDH assay was carried out to assess the viability of the tissue. The viability remained above 70% in all the time points and tested groups, therefore the treatments proved their biocompatibility on the ex vivo model, as illustrated on Figure 5A.

Although there was no cytotoxicity, significant differences were observed among groups. On day 4, the viability was about 90% in all the groups, being the highest in the control group and the lowest in the group treated with bHF+Chit (*p > 0.05). On day 8, the differences among groups were more pronounced (***p < 0.001). The viability of the groups treated with bHF+Chit and mHF-Lac, decreased to values around 70% and 80%, respectively, while the viability of the control group was maintained above 90%.

Wound closure was evaluated measuring the epithelial gap on H&E stained tissue sections, as observed in Figure 5B,C. In the group treated with mHF-Lac a slight acceleration in wound closure was observed, although it was not statistically significant (Figure 5D).

**Figure 6:**
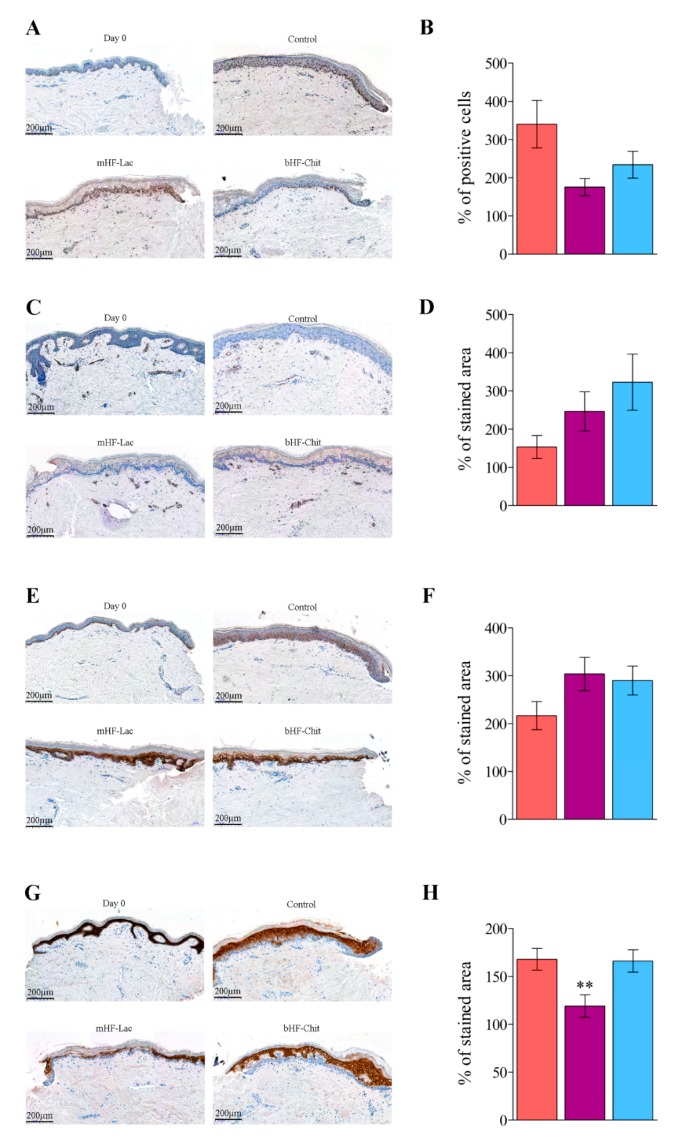
(**A**) Representative images of tissue sections stained against PCNA. (**B**) Representation of PCNA positive cells percentage regarding to baseline. (**C**) Representative images of tissue sections stained against α-SMA. (**D**) Representation of quantitative analysis of α-smooth muscle actin (α-SMA) stained area. (**E**) Representative images of tissue sections stained against cytokeratin 14. (**F**) Representation of quantitative analysis of cytokeratin-14 stained area. (**G**) Representative images of tissue sections stained against cytokeratin 10. (**H**) Representation of quantitative analysis of cytokeratin-10 stained area. **p > 0.001 comparing the group treated with the mHF-Lac and the other two groups on day 8. Scale bar on figures A, C, E and G indicates 200 µm. Results on images B,D,F and H are shown as the mean ± standard error of the mean.

In addition, immunohistological analyses were performed. Tissue sections were stained against PCNA (Figure 6A), a marker for cellular proliferation, which gives an insight about the cellular proliferation into the wound [29]. In order to decrease the interindividual variability, results were represented as the percentage of positive cells in regards to the baseline of each patient. Results depicted in Figure 6B showed non-significant differences in cell proliferation.

Wound contraction was analysed using antibodies against α-SMA, a marker of myofibroblast differentiation, as shown in Figure 6C [30]. In keeping with the rest of the results, after analysing the percentage of stained area, no differences were found among the groups (Figure 6D).

The expression of two cytokeratins was also evaluated, cytokeratin 14 and 10, precisely. Cytokeratin 14 is a marker for stratifying keratinocytes into the epithelial tongue. Cytokeratin 10, on the contrary, is a marker for differentiated keratinocytes, which is not found on the epithelial tongue, just in the mature epithelium [28,31,32]. The analysis of the stratifying keratinocytes showed similar cytokeratin 14 expression in all the groups (representative images on Figure 6E and quantitative analysis on Figure 6F). The images and results obtained in the analysis of cytokeratin 10 expression are illustrated on Figure 6G,H, respectively. Similar values were achieved on the control group and the group treated with bHF+Chit, while a smaller stained area was observed on the group treated with mHF-Lac (**p < 0.01).

## 4. Discussion

The aim of the current study was to develop a bioinspired bilayer dressing composed of gelatin. In order to mimic the natural structure of the skin, different crosslinkers were used for each gelatin layer, thus conferring them unique characteristics. The upper layer was composed of gelatin crosslinked with lactose (mHF-Lac) to obtain a resistant and non-hydrolytically labile layer. Lactose and gelatin reacted in presence of heat leading to a non-enzymatic glycation of the protein chains known as Maillard reaction [33]. The hydrofilm developed using this procedure was previously characterised by our research group [26]. It consisted of a homogeneous and transparent layer of about 50 µm thickness that maintained its initial appearance after being immersed in PBS at 37 °C or incubated with cells for 8 days.

The lower layer was composed of gelatin crosslinked with citric acid (mHF-CA), giving rise to the reaction of the acid with the primary amines of the gelatin’s protein chains [13]. This hydrofilm presented a more jelly-like structure, as it was able to absorb a considerably larger volume of water, losing their integrity after a few hours immersed in PBS at 37 °C (data not shown). The influence of the crosslinker on the gelatin structure and characteristics was demonstrated by FTIR results, showing that mHF-Lac presented a progressive conversion of residual regular structures and unordered segments into intermolecular β-sheets. As also shown by SEM analysis, this lactose mediated change in the secondary structure of gelatin led to a more compact and smoother microstructure than that obtained by citric acid addition.

Chitosan was added to the lower layer in order to promote wound healing process (mHF-CA+Chit), as chitosan has previously demonstrated various beneficial effects in this sense. In fact, it presents antibacterial and hemostatic activity, acting in the early phases of healing [15]; it promotes the activity of macrophages and the migration of polymorphonuclear neutrophils [16]; and it is able to induce the proliferation of fibroblasts and keratinocytes both in vitro and in vivo [34,35]. In addition, it regulates in a biphasic way the expression of TGFβ1, upregulating it during the early healing phases (increased collagen production); while downregulating it at the later phases (reduced the scar formation) [36].

To obtain bilayer hydrofilms (bHF and bHF+Chit), both layers were glued together by spraying ethanol on them and letting them air dry (mHF-Lac with mHF-CA and mHF-Lac with mHF-CA+Chit, respectively).

The swelling ability of the resulting hydrofilms was evaluated in order to compare the behaviour of the different formulations. Assays were conducted at 4 °C instead of at skin temperature, given that mHF-CA and mHF-CA+Chit lost their structural stability in a warm aqueous environment. The water uptake of gelatin varies depending on the hydrofilm microstructure, which could be related with the gelatin crosslinking [37,38]. On the one hand, amino groups of lysine residue in gelatin reacts with the carbonyl group of lactose through Maillard reaction, which is known to lead to compact structures [39], decreasing the capacity of the hydrofilms for water uptake. On the other hand, citric acid has three carboxyl groups with different reactivity and, thus, those unreacted carboxylic groups can physically interact with the polar groups of gelatin through hydrogen bonding, leading to looser structures, increasing the swelling ability of the hydrofilms. Therefore, the hydrofilms with the most compact structure (mHF-Lac) had the lowest ability to absorb water, and the ones with less compact structure (m-HF-CA and m-HF-CA+Chit) had the highest water uptake. Accordingly, bilayer hydrofilms had intermediate values, since they were composed of both layers.

The hydrolytical stability study was also carried out at 4 °C to extend the study time and allow a better comparison of the characteristics of the different formulations. All the hydrofilms maintained about the 96% of their dry weight after being immersed in PBS for 72 h. This weight loss may be due to the dissolution of the unreacted crosslinker, as described previously in gelatin films crosslinked with lactose [40].

The occlusivity of the hydrofilms was evaluated measuring the WVTR, in order to assess if they were able to maintain an acceptable level of moisture into the wounds. The WVTR of the tested hydrofilms was within the range of commercial wound dressings (426–2047 g/m^2^·day). Thus, they presented an adequate control of moisture, allowing exudate to evaporate, while maintaining some moisture into the wound to prevent tissue dehydration. This helps in re-epithelisation and angiogenesis, and also relieves pain [41,42,43]. The higher permeability of the mHF-Lac in comparison to the bilayer hydrofilms may be explained by the lower thickness of the former, as previously reported [44].

Prior to evaluating the efficacy of the dressings ex vivo, their cytocompatibility was assessed. The results obtained in the indirect cytotoxicity assay with all the hydrofilms tested showed viability values above 70% with respect to the control group, suggesting an excellent biocompatibility according to the ISO 10992-5:2009 guidelines for biological evaluation of medical devices. The direct cytotoxicity assay included in this guidelines was not performed, since gelatin crosslinked with citric acid partially dissolves at 37 °C, thereby increasing the viscosity of the medium and impeding the removal of the hydrofilms to perform the CCK-8 assay. The viability assay demonstrated that there was no need of processing the hydrofilms through dialysis, as similar viability results were obtained hydrating them for 15 min prior to the assay. Moreover, formulations containing chitosan (mHF-CA+Chit and bHF+Chit) achieved a higher viability after hydration than after dialysis, probably because some of the chitosan was lost during the dialysis process, and thus hydrated hydrofilms contained a higher amount of chitosan which had shown to promote fibroblasts proliferation [34]. Thereby, a rapid conditioning step of 15 min hydration was enough in order to remove the unreacted crosslinker.

Dressings efficacy was evaluated using an ex vivo assay in human skin. This model was chosen over animal models in order to minimize the differences in the healing process and skin structure between humans and animals. [17]. In addition, this ex vivo method reduces the use of animals in experimentation following the principle of the 3R [45]. Although it is not standardized yet, ex vivo human wound models have been already used to evaluate the short-term effect on wound healing of different dressings and formulations [27,46,47,48,49,50]. However, the use of these model is not very expanded, due to the technical complexity and the limited availability of human skin explants [51]. Another major limitation of this method is the interindividual variability, due to the differences among patients, which can be overcome normalizing the results to the control in each patient. Furthermore, the viability of the explanted tissue limits the duration of the studies to 7–14 days, enabling only short-term evaluation of wound healing. Finally, during physiological wound healing a massive recruitment of inflammatory cells from blood occurs, which cannot happen in ex vivo models due to the lack of blood circulation.

In our case, a major limitation of the model was the impossibility to conduct effective full thickness wounds, since the surrounding dermal tissue was introduced into the wounds right after the transferal of the biopsies into the culture inserts. Whereby the model was considered a partial wound model. A way to avoid the dermis expansion into the wound is to fill the void with autologous fat or the scaffold itself, subsequently to wound induction [27,47]. However, in this study, instead of inserting the treatments inside the wounds, dermis was allowed to expand, filling partially the wounds. Thereafter, hydrofilms were applied on top of them, in order to let the lower layer of the hydrofilms adapt to the wound bed composed of the expanded dermis, due to its great moldability and swelling ability, while the upper layer was maintained outside, protecting the wounds.

Wet hydrofilms were applied on top of the biopsies, as they were very moldable they adapted to the surface of the biopsies. In addition, due to the great swelling ability of the lower layer, it absorbed fluid, filling completely the void of the wound, and thus, being in close contact with the wound bed. Thereafter, hydrofilms were changed on day 4, since in a clinical environment, diabetic wounds are usually cleaned every 1–3 times a week [52].

In order to evaluate the viability of the biopsies, a LDH assay was conducted on days 4 and 8. All groups presented viability values above 70%. On one hand, those values demonstrated that cells of the biopsies remained alive during the whole study. On the other hand, this study proved the biocompatibility of the developed hydrofilms, which is in keeping with the previously observed in vitro cytotoxicity assay carried out in fibroblasts. Moreover, assessing the viability assay into the ex vivo model gives an insight of the behaviour of the hydrofilms in a more complex scenario than a monolayer single cell culture.

In addition, the wound healing process was assessed to analyse the effect of the hydrofilms. In general, the application of the hydrofilms did not hinder healing, since no differences were observed among groups in the majority of the parameters evaluated, such as wound closure, cellular proliferation, wound contraction and expression of new undifferentiated keratinocytes.

Overall, in this study bilayer hydrofilms were developed through a simple and cost-effective process. One of the main novelties lies in the use of two non-toxic and innovative crosslinkers, lactose and citric acid, which have not been used in wound healing applications yet. The combination of these crosslinkers allowed us to obtain two well differentiated layers of gelatin to cover the needs of an ideal wound dressing. Thus, the upper layer provides mechanical and hydrolytic stability (protection), while the layer below is able to swell and maintain suitable moisture conditions (among other benefits) required for wound closure.

Finally, the results obtained in the ex vivo assay showed the suitability of the model to assess wound healing, especially as a screening method prior to conduct an in vivo study. In addition, it showed that the developed hydrofilms might be useful as wound dressings, as they had demonstrated to be biocompatible and not to hamper the healing process. These results open the door to new studies where bioactive molecules, such as growth factors, will be encapsulated or immobilised into the lower layer with the aim of improving the efficacy of the dressings.

## 5. Conclusions

In the current study, a bioinspired gelatin and gelatin/chitosan bilayer wound dressing was developed. Firstly, each layer separately and the resulting bilayer hydrofilms were characterised, showing that the upper layer (mHF-LAc) was more resistant and that the lower layer (mHF-CA and mHF-CA+Chit) had a greater ability to absorb water. Overall, the dressings presented good swelling and occlusivity characteristics and they did not show cytotoxicity in vitro. In order to evaluate its biocompatibility and efficacy, an ex vivo wound healing assay was performed. Results demonstrated that the bioinspired hydrofilm was well tolerated, achieving similar results to the control in terms of both biocompatibility and wound healing. This may be a good starting point to continue developing more sophisticated wound dressings based on this bioinspired bilayer hydrofilm, primarily by including bioactive molecules in the lower layer.

## Figures and Tables

**Table 1:** Summary of the developed monolayer hydrofilms (mHF) and bilayer hydrofilms (bHF) based on gelatin.

Name	Mono Bilayer Hydrofilm	Crosslinking Agent		Chitosan Addition
		Upper Layer	Lower Layer	
mHF-Lac	Monolayer	Lactose		No
mHF-CA	Monolayer	Citric acid		No
mHF-CA+Chit	Monolayer	Citric acid		Yes
bHF	Bilayer	Lactose	Citric acid	No
bHF+Chit	Bilayer	Lactose	Citric acid	Yes

**Table 2:** Demographic data and source of explants.

Patient Number	Gender	Age (Years)	Anatomical Source of Skin
1	Female	50	Abdomen
2	Male	60	Abdomen
3	Female	49	Breast
